# Women’s Satisfaction with Breastfeeding and Risk of Exclusive Breastfeeding Interruption

**DOI:** 10.3390/nu15245062

**Published:** 2023-12-11

**Authors:** Agnes Meire Branco Leria Bizon, Camila Giugliani, Elsa Regina Justo Giugliani

**Affiliations:** 1 Programa de Pós-Graduação em Saúde da Criança e do Adolescente, Faculdade de Medicina, Universidade Federal do Rio Grande do Sul (UFRGS), Porto Alegre 90035-003, RS, Brazil; 2 Programa de Pós-Graduação em Epidemiologia, Faculdade de Medicina, Universidade Federal do Rio Grande do Sul (UFRGS), Porto Alegre 90035-003, RS, Brazil; cgiugliani@hcpa.edu.br; 3 Hospital de Clínicas de Porto Alegre, Porto Alegre 90035-003, RS, Brazil

**Keywords:** exclusive breastfeeding, maternal and child health, personal satisfaction

## Abstract

This prospective cohort study was conducted to evaluate the association between women’s satisfaction with breastfeeding at 1 month post-partum and the risk of exclusive breastfeeding (EBF) interruption before 6 months. 287 mother–infant dyads randomly selected from two maternity hospitals were followed from birth to 24 months of infant’s age. Women’s satisfaction with breastfeeding was assessed using the Maternal Breastfeeding Evaluation Scale (MBFES) at 1 month. The association between women’s satisfaction with breastfeeding and risk of EBF interruption before 6 months was estimated using Cox proportional hazards model. Kaplan–Meier survival curves for EBF were compared between women with lower satisfaction with breastfeeding (MBFES score < median 124) and those with higher satisfaction (MBFES score ≥ 124). Median EBF duration in women with higher satisfaction was 120 days (95%CI 109–131), vs. 26 days (95%CI 19–33) in less satisfied women. Each additional point on MBFES promoted a reduction of 2.0% in the risk of EBF interruption. Among women with satisfaction scores < 124, the risk of EBF interruption was 86% higher when compared with those ≥ 124 (adjusted hazard ratio 1.86; 95%CI 1.41–2.46). Lower maternal satisfaction with breastfeeding in the first month post-partum is associated with a higher risk of EBF interruption before 6 months.

## 1. Introduction

Despite the large body of evidence supporting the positive impact of breastfeeding on both child and maternal health, this way of feeding a small infant is still too seldom practiced [1]. At the global level, 48% of infants younger than 6 months are exclusively breastfed, 70% are breastfed at 1 year, and 45% at 2 years [2]. In Brazil, these breastfeeding indicators are below the global average: 45.8%, 52.1% and 35.5%, respectively [3]. Both globally and in Brazil, much effort will be needed to achieve the WHO/UNICEF 2030 targets for exclusive breastfeeding (70%), continued breastfeeding up to at least 1 year (80%), and continued breastfeeding up to at least 2 years (60%) [2].

The identification of factors that can influence breastfeeding duration—especially modifiable ones—is paramount for the planning and implementation of interventions, especially in populations which are most vulnerable to early interruption of breastfeeding. Among the modifiable factors that deserve investigation, women’s satisfaction with breastfeeding has become increasingly valuable, since it involves aspects that are often neglected by health professionals, such as expectations, desires, and the needs of breastfeeding women and their infants, the bonding between mother and child, as well as the woman’s self-confidence as a mother [4,5,6,7].

Breastfeeding success is often assessed by researchers and health professionals based on its duration or on the absence of problems. Nevertheless, some studies have shown that, from the woman’s point of view, the quality of the breastfeeding experience seems to be as important as or even more important than the duration or exclusivity of breastfeeding [5,6,7,8,9,10]. Still, women’s satisfaction with breastfeeding has been given little value so far.

The first studies on the relationship between maternal satisfaction with breastfeeding and the duration of this practice emerged in the 1990s. Since then, two studies have shown a weak correlation between women’s satisfaction with breastfeeding and its duration [8] and one found a strong correlation [10]. Two other studies showed a positive association between them [5,6]. Studies evaluating the influence of women’s satisfaction on exclusive breastfeeding duration are scarce [10,11] and none have evaluated the influence of maternal satisfaction with breastfeeding in the first month after birth on the practice of exclusive breastfeeding throughout the first 6 months of the infant’s life. Thus, considering the importance of exclusive breastfeeding and the scarcity of studies exploring women’s satisfaction with breastfeeding, especially in Brazil, the present study aimed to evaluate the association between women’s satisfaction with breastfeeding at 1 month post-partum and the risk of exclusive breastfeeding interruption throughout the first 6 months. These findings might be useful in promoting, protecting and supporting breastfeeding, at both the individual and collective levels, not only to improve indicators, but also to enhance the quality of women’s breastfeeding experience.

## 2. Materials and Methods

In this prospective cohort study conducted in the municipality of Porto Alegre, southern Brazil, mother–infant dyads were followed for 24 months. The sample comprised mothers who gave birth at two large maternity hospitals in the city, one public and one private. In 2016, these two maternity hospitals accounted for 3725 and 4182 deliveries, respectively, of a total of 30,268 [12,13]. In order to be included in the study, mothers and their respective newborns should meet the following criteria: residing in the municipality at the time of delivery; singleton, live full-term newborn (gestational age ≥ 37 weeks); mother and infant staying together in the same room (rooming-in) during the hospital stay; and having initiated breastfeeding. Exclusion criteria consisted of any problem observed in the mother or newborn that could significantly affect breastfeeding, e.g., orofacial malformations or any serious illness that required separation between mother and newborn. Dyads residing in areas with high rates of violence (defined as areas where primary health care worker visits were suspended for security reasons) were also excluded in order to preserve the safety of the interviewers.

A sample size of 219 women was calculated for this study’s objective, using the WinPepi version 11.43, considering the following parameters: significance level of 5%, power of 80%, 20% of participants lost to follow-up, and a difference of 20 percentage points in the rates of exclusive breastfeeding in infants younger than 6 months between women with higher vs. lower levels of satisfaction, according to data found in the literature [14]. In order to reflect the public vs. private distribution in health service utilization in Brazil (approximately 70% and 30%, respectively [15]), we projected the selection of one woman at a private maternity hospital for every two women at the public facility.

Women were selected daily, also on the weekends, between January and July 2016, at the rooming-in section of the obstetric units. Dyads meeting the inclusion criteria and who had given birth in the past 24 h were considered eligible. Subsequently, the eligible dyads were randomly selected for inclusion using the lottery method. If the initially selected dyad was found to meet an exclusion criterion, or refused to participate, that dyad was replaced by repeating the lottery draw procedure.

The dyads were followed from birth to 24 months of infant’s age, or to the age of weaning if before 24 months. For the present study, only the data relating to the infant’s 6 months of life were used.

Interviews were conducted by 10 interviewers, all working in the health field and previously trained for the task. The first contact was made at the maternity ward, where women were invited to participate in the study; those who agreed were asked to answer a brief questionnaire covering demographic data and delivery information. Between 31 and 37 days after birth, the first home visit took place. At this occasion, a standardized questionnaire was administered to obtain data on socio-demographic characteristics, woman’s health, information on latest pregnancy, delivery and immediate post-partum period, and the first month of life of the infant. Subsequent contacts were made by telephone at 2 and 4 months, and in person during a home visit at 6 months, to obtain updated information on the infant’s feeding habits. Dyads who were not found after three telephone contact and one home visit attempts were considered as losses. Whenever dyads were lost to follow-up at some point of the data collection process, attempts were made to interview these women at subsequent data collection points.

Women’s satisfaction level with breastfeeding in the first month post-partum was the main variable of interest. The information was obtained via self-application of the Maternal Breastfeeding Evaluation Scale (MBFES) [16] during the first home visit. This instrument assesses maternal perception of the quality of their breastfeeding experience, considering not only maternal factors, but also child factors. The original version of the MBFES [17] is comprised of 30 items, distributed into three subscales: maternal pleasure and role, child satisfaction and growth, and maternal lifestyle and body image. For each item, there are 5 Likert-type answers, ranging from 1 point (totally disagree) to 5 points (totally agree); higher values indicate higher satisfaction levels. The MBFES used in the present study was validated for use in the Brazilian population [16] starting from the version translated and validated into Portuguese by Galvão [6]. The Brazilian version maintained the three subscales of the original instrument, but resulted in 29 items, out of the original 30, due to the low factor loading of one item. Thus, the total possible score could range from 29 to 145. The validation process of the Brazilian version of the MBFES showed that it is a valid and reliable tool to be applied to the Brazilian population (Cronbach’s alpha = 0.88, 95%CI 0.86–0.90). More details of the Brazilian Portuguese validation process can be found elsewhere [16].

The outcome of this study was defined as the interruption of exclusive breastfeeding before 6 months of infant’s age, measured as days of exclusive breastfeeding. Exclusive breastfeeding was defined according to the WHO criteria, i.e., receiving breast milk, either directly from the mother’s breast, extracted from the mother’s breast, or human donor breast milk, with no other liquid or solid foods, not even water, except for drops or solutions containing vitamins, oral rehydration salts, mineral supplements or medicine [18].

Statistical analyses were performed using the Statistical Package for the Social Sciences (SPSS) for Windows version 21.0 (IBM, Chicago, IL, USA). Using the chi-square test, the group of women who concluded the study were compared to those who were excluded, to those who refused to participate, and also to those who were lost to follow-up. Results showing *p* ≤ 0.05 were considered significant.

Kaplan–Meier survival curves were calculated to illustrate the time to interruption of exclusive breastfeeding among women presenting lower satisfaction with breastfeeding (MBFES score below the median) vs. those with higher satisfaction levels (MBFES score at or above the median). The survival analysis was also used to calculate the median duration of exclusive breastfeeding and to assess the accumulated probability of exclusive breastfeeding duration.

The association between women’s satisfaction with breastfeeding and risk of exclusive breastfeeding interruption before 6 months was estimated as hazard ratios and respective 95% confidence intervals (95%CI) using the Cox proportional hazards multivariate regression model. The explanatory variable was used in two different ways: via the MBFES score obtained continuously, and via the median obtained with application of the instrument (124 points).

Variables added to the adjustment model were those reaching *p* ≤ 0.2 in the univariate analysis. Categories used as reference were those known to protect exclusive breastfeeding, according to information from the literature. The following variables were explored: socio-demographic characteristics of the woman (age, socio-economic level, schooling level, skin color, parity, and cohabitation with infant’s father), infant’s sex, data related to hospital care (type of birth and type of hospital (public vs. private)), and breastfeeding problems in the first month post-partum (breast engorgement, pain while breastfeeding, cracked nipples, perceived low milk supply, infant difficulties with latching on/sucking). Socio-economic level was divided in five strata, A (better off) to E, according to criteria proposed by the Brazilian Association of Research Companies [19]. The effect measure was considered to be modified when *p* ≤ 0.05.

To control for data quality, answers given to key questions of the questionnaire were checked in approximately 5% of the sample, via telephone contact, concomitantly with data collection, at all data collection stages.

The present study was conducted in line with norms and regulations applicable to research involving humans (Resolution 466/2012 of the Brazilian National Health Council) and was approved by the Ethics Committees of Hospital de Clínicas de Porto Alegre and Hospital Moinhos de Vento (CAAE 49938015.3.0000.5327 and 46775115.0.3002.5330). All women who agreed to participate in the study signed an informed consent form before the start of data collection.

## 3. Results

Of the 503 women selected for the study using the lottery method, 124 were excluded because they lived in areas with high rates of violence, and 25 (5%) refused to participate in the study. The characteristics of the excluded women did not differ from those of the women who participated in the study with regard to skin color (*p* = 0.949), parity (*p* = 0.384), age (*p* = 0.286) and infant’s sex (*p* = 0.746); however, the women excluded showed lower schooling level (*p* < 0.001) and a higher prevalence of vaginal deliveries (*p* = 0.01). Conversely, the profile of the women who refused to participate was similar to that of the women who participated in the study with regard to skin color (*p* = 0.125), parity (*p* = 1.00), and age (*p* = 0.279), but they differed by presenting lower schooling level (*p* < 0.001).

In addition, 67 women could not be located for the first home visit interview. These women showed differences in relation to the women who participated in the study with regard to schooling level and skin color—they showed lower schooling level (none had started college vs. 43.2%; *p* < 0.01) and a higher prevalence of white skin color (87.7% vs. 75.3%; *p* = 0.032). A total of 30 (10.4%) women were lost to follow-up, i.e., interviewed at the end of the first month but not found for the interview at 6 months.

After the losses and excluding women who interrupted breastfeeding before 6 months, the number of mother–infant dyads included in the study at each data collection stage was as follows: 287 at 30 days, 228 at 60 days, 218 at 120 days, and 213 at 180 days.

Maternal age ranged from 16 to 45 years, with a mean of 29 years (standard deviation ± 6.6). Most women had white skin color, lived with the infant’s father, and did not have a college degree. The following variables were associated with exclusive breastfeeding duration in the univariate analysis (*p* ≤ 0.2) and were therefore included in the multivariate model: maternal age and schooling level, cohabitation with infant’s father, and breastfeeding problems in the first 30 days post-partum, namely, cracked nipples, perceived low milk supply, and infant difficulties with latching on/sucking. The socio-economic variable was also associated with the outcome, but was not added to the multivariate analysis due to its strong interaction as a proxy for schooling level (Table 1).

The median MBFES score in the sample was 124, with an interquartile range of 113 to 131. The median duration of exclusive breastfeeding in the whole sample was 67 days (95%CI 41–93), i.e., 120 days (95%CI 109–131) among women with higher levels of satisfaction (score at or above the median) and 26 days (95%CI 19–33) among the women with lower satisfaction (score below the median). This difference was statistically significant (*p* < 0.001).

**Figure 1:**
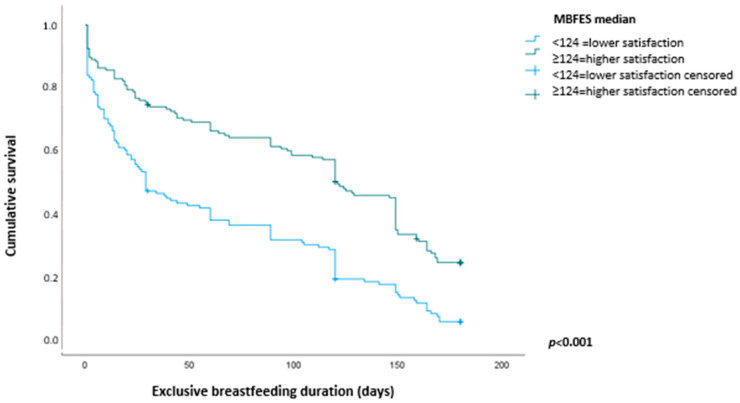
Survival curves for exclusive breastfeeding duration, considering woman’s satisfaction with breastfeeding. Porto Alegre, Brazil, 2018. MBFES = Maternal Breastfeeding Evaluation Scale.

Figure 1 shows Kaplan–Meier survival curves calculated for exclusive breastfeeding over the first 6 months of infant’s age according to women’s satisfaction levels with breastfeeding (higher vs. lower levels) assessed at 30 days of life of the infant.

A positive association was found between exclusive breastfeeding duration and MBFES score at 30 days: for each additional point in the score, a reduction of 2.0% was observed in the risk of exclusive breastfeeding interruption. Among women with lower levels of satisfaction (score < 124), the risk of exclusive breastfeeding interruption was 86% higher when compared with women showing higher satisfaction levels (scoring ≥ 124; Table 2).

Table 3 presents the accumulated probability of exclusive breastfeeding over the first 6 months of life of the infant, according to maternal satisfaction with breastfeeding. The data show that the risk of breastfeeding interruption among women with lower satisfaction levels increases over time.

## 4. Discussion

The present study showed that women with lower levels of satisfaction with breastfeeding in the first month of life of the infant, as assessed by the MBFES, were at an increased risk of interrupting exclusive breastfeeding before 6 months. For every additional point in the MBFES score, that risk decreased by 2.0%. The risk of exclusive breastfeeding interruption was 86% higher among women scoring below the median when compared to women with higher scores. Particularly interesting is the fact that, throughout the period assessed, lower levels of satisfaction with breastfeeding in the first month increased the risk of exclusive breastfeeding interruption at all subsequent months, suggesting that maternal satisfaction with breastfeeding in the first month post-partum can be an indicator of risk of exclusive breastfeeding interruption over the first 6 months post-partum.

There are already some studies pointing to an association between women’s satisfaction with any breastfeeding and its duration. Among the most significant is the study by Galvão [6], carried out with Portuguese women, which showed a close relationship between satisfaction with breastfeeding measured at different times and the duration of any breastfeeding. Yet studies on breastfeeding satisfaction focusing on exclusive breastfeeding are rare. One of them is a longitudinal, quasi-experimental study, conducted in Australia [10]. That study found a strong positive correlation between maternal perception of breastfeeding success, assessed using MBFES, and exclusive breastfeeding length (r = 0.63; *p* < 0.001). The other study, conducted in Poland, assessed maternal satisfaction with breastfeeding at 3 months of the infant’s life, using a scale from 0 to 10, with 10 being the best level. The authors concluded that maternal satisfaction with breastfeeding was one of the predictors of exclusive breastfeeding at 6 months (adjusted OR (95%CI) 1.44 [1.01–2.06], *p* = 0.04) [11]. The results of those two studies are in line with the results of the present study, corroborating the conclusion that women with lower breastfeeding satisfaction levels breastfeed exclusively for shorter times.

The association between lower woman satisfaction with breastfeeding and higher risk of exclusive breastfeeding interruption before 6 months found in this study is not surprising—women who are more satisfied with breastfeeding are expected to breastfeed for longer. However, demonstrating that association is very important, as it adds one further element to the discussion when evaluating risk of early exclusive breastfeeding interruption. When low levels of maternal satisfaction with breastfeeding are detected, interventions should be discussed with the woman to improve her satisfaction, not only in an attempt to increase exclusive breastfeeding duration (if wished by the mother), but also to make the unique breastfeeding experience with the child a more enjoyable one.

The level of satisfaction with breastfeeding observed among the women assessed in this study was high (median of 124 points from a maximum of 145). In Australia, the mean scores obtained with MBFES for maternal satisfaction with breastfeeding were 116 at 15 days post-partum and 117 at 45 days [5]. In the present study, women seemed more satisfied than in the Australian study, especially if we take into consideration that the maximum score of the original instrument is 150 (not 145, as in the Brazilian version). Conversely, the median found in Portuguese women assessed at 1 and 6 months of infant’s age was 133 [4], higher than the median in the present investigation.

The significant difference observed in the median exclusive breastfeeding duration among women with higher satisfaction when compared to those with lower satisfaction levels, namely, 120 vs. 26 days, stands out. Certainly, a difference of 3 months in exclusive breastfeeding duration will have consequences to the infant’s health. A study conducted in the United Kingdom found an estimated reduction of 53% in hospitalizations due to diarrhea and of 27% due to respiratory tract infections for every month of exclusive breastfeeding [20].

Although this study has shown an association between women’s level of satisfaction with breastfeeding and duration of exclusive breastfeeding, it did not explore the reasons for more or less satisfaction. It is known that post-partum depression may increase the risk of early interruption of breastfeeding [21]; on the other hand, weaning can induce or worsen depression [22]. It is very likely that maternal satisfaction with breastfeeding is involved in this association. In fact, the interrelationship between satisfaction with breastfeeding and symptoms of post-partum depression has already been demonstrated [23]. Therefore, in addition to assessing the women’s satisfaction with breastfeeding, it is important and necessary to obtain information about the mother’s psychosocial environment, including information on her support network, and the mental health of her companion and other close relatives.

Among the strengths of this original study, we highlight its methodology: a cohort study with a randomly selected sample, followed for 24 months, rigorously conducted with face to face interviews, use of an instrument validated for the Brazilian population (MBFES) for the assessment of woman satisfaction with breastfeeding, not to mention the quality control on data collected throughout the follow-up period. The frequency of interviews conducted in the first 6 months of infant’s age (at 1, 2, 4, and 6 months) virtually eliminated any memory bias with regard to exclusive breastfeeding duration.

Notwithstanding, the study also presents some limitations. One of them is the exclusion of women who resided in more violent areas, which may have affected the external validity of the findings. Still, comparison of those women to those who completed the study showed that both groups were similar in most of the variables analyzed, differing only with regard to schooling level and type of delivery. This limitation was minimized by including schooling level in the multivariate model; type of delivery was not included due to the absence of a significant association with the outcome in the univariate analysis. Another limitation was the number of participants lost to follow-up, but we believe that the statistical model employed has helped minimize that bias as well. The differences between the group of women who were lost to follow-up and those who completed the study (schooling level and skin color) may have interfered with the estimated values, but not with the association found between women’s satisfaction with breastfeeding in the first month post-partum and duration of exclusive breastfeeding.

## 5. Conclusions

The present study confirmed the existence of an association between women’s lower satisfaction with breastfeeding at the first month post-partum and an increased risk of exclusive breastfeeding interruption before 6 months of infant’s age. Given the importance of exclusive breastfeeding for the mother–infant dyad, in terms of health benefits and well-being, this study makes a relevant contribution by highlighting the central role played by mother’s satisfaction with breastfeeding, as well as the need to assess it in clinical practice, especially in the first month post-partum. Once the health professional becomes aware of the low levels of satisfaction reported by a breastfeeding woman, and discusses with her the factors that might be contributing to this, it becomes possible to propose interventions, with the aim not only of postponing weaning, but especially of promoting a more enjoyable experience for the mother–infant dyad. This is an important contribution for the practice of primary health care workers, clinical breastfeeding consultants, and community midwives. At the collective level, this study suggests that interventions to increase maternal satisfaction with breastfeeding could be useful as part of breastfeeding promotion, protection and support actions, with the goal of increasing the rates of exclusive breastfeeding in infants younger than 6 months, keeping in mind the WHO’s and UNICEF’s goal to reach 70% by 2030 [2]. Further similar studies should be conducted with other populations to confirm the present findings. Likewise, studies looking in more detail at the factors associated with higher or lower satisfaction with breastfeeding, and at the factors involved in this association, are also warranted. Such knowledge could then help health professionals prevent and also better handle maternal dissatisfaction with breastfeeding.

## Figures and Tables

**Table 1:** Sample characteristics (n = 287). Porto Alegre, Brazil, 2016.

Variable	n = 287	%	Exclusive Breastfeeding Interruption ^a^ *p* ^b^
Age			
<30 years	142	49.5	0.074
≥30 years ^e^	145	50.5	
School level (college)			
Yes ^e^	100	34.8	0.016
No	187	65.2	
Socio-economic classification			
A/B ^e^	163	56.8	0.233
C/D/E	122	42.5	
Lost	2	0.07	
Skin color			
White	216	75.3	0.554
Black/brown ^e^	71	24.7	
Parity			
Primiparous	142	49.5	0.424
Multiparous ^e^	145	50.5	
Cohabitation with infant’s father			
Yes ^e^	248	86.4	0.007
No	39	13.6	
Return to work ^c^			
Yes	142	49.5	0.220
No ^e^	145	50.5	
Hospital type			
Public ^e^	194	67.6	0.706
Private	93	32.4	
Type of delivery			
Vaginal ^e^	149	51.9	0.995
C-section	138	48.1	
Newborn sex			
Male ^e^	136	47.4	0.814
Female	151	51.6	
Breast engorgement ^d^			
Yes	134	46.7	0.860
No ^e^	153	53.3	
Pain while breastfeeding ^d^			
Yes	182	63.4	0.328
No ^e^	105	36.6	
Cracked nipples ^d^			
Yes	135	47.0	0.028
No ^e^	152	53.0	
Perceived low milk supply ^d^			
Yes	84	29.3	<0.001
No ^e^	203	70.7	
Difficulties latching on/sucking ^d^			
Yes	63	22.0	<0.001
No ^e^	224	78.0	

^a^ Exclusive breastfeeding interruption before 6 months of infant’s age. ^b^ Cox regression to test variables included in the multivariate model (*p* ≥ 0.20). ^c^ Return to work at any time in the first 6 months post-partum. ^d^ Breastfeeding problems in the first 30 days post-partum. ^e^ Reference category.

**Table 2:** Cox proportional hazards multivariate regression model to test the association between risk of exclusive breastfeeding interruption before 6 months of infant’s life and women’s satisfaction with breastfeeding in the first month post-partum (n = 287). Porto Alegre, Brazil, 2018.

	Univariate Analysis		Multivariate Analysis ^a^	
Model	HR (95%CI)	*p*	HR (95%CI)	*p*
Risk of exclusive breastfeeding interruption × MBFES score	0.97 (0.96–0.98)	<0.001	0.98 (0.97–0.99)	<0.001
Risk of exclusive breastfeeding interruption × lower satisfaction with breastfeeding ^b^	2.15 (1.66–2.78)	<0.001	1.86 (1.41–2.46)	<0.001

95%CI = 95% confidence interval; HR = hazard ratio; MBFES = Maternal Breastfeeding Evaluation Scale. ^a^ Adjustment variables: maternal age, maternal schooling level, cohabitation with infant’s father, and breastfeeding problems: cracked nipples, perceived low milk supply, and difficulty latching on/sucking. ^b^ Median MBFES score: 124.

**Table 3:** Cumulative probability of risk of exclusive breastfeeding interruption along the infant’s first 6 months of age according to maternal satisfaction with breastfeeding measured at 30 days post-partum. Porto Alegre, Brazil, 2018.

Time	Exclusive Breastfeeding Probability (%) ^a^			Exclusive Breastfeeding Interruption before 6 Months, HR ^b^ (95%CI) ^c^	*p*
	Total Id	MBFES < 124 Id	MBFES ≥ 124 Id		
30 days	58.9	43.6	73.5	1.68 (1.09–2.57)	0.018
60 days	50.4	34.9	65.2	1.64 (1.11–2.43)	0.013
90 days	45.1	29.0	60.3	1.76 (1.24–2.51)	0.002
120 days	33.7	17.4	49.2	1.73 (1.23–2.44)	0.002
150 days	22.8	12.7	32.6	1.73 (1.29–2.32)	<0.001
180 days	14.5	4.8	23.7	1.86 (1.41–2.46)	<0.001

95%CI = 95% confidence interval; HR = hazard ratio; Id = incidence density; MBFES = Maternal Breastfeeding Evaluation Scale. ^a^ Percentages were obtained using the Kaplan–Meier method. ^b^ Adjusted for the following variables: maternal age and schooling level, cohabitation with infant’s father, occurrence of cracked nipples, perceived low milk supply, and difficulty latching on/sucking. ^c^ 95%CIs obtained with Cox proportional hazards multivariate regression model.

## Data Availability

The datasets used and analyzed in this study are available from the corresponding author on request.
